# Possible mechanisms of treatment for spinal cord injury repair with tanshinone IIA

**DOI:** 10.3389/fnmol.2023.1277755

**Published:** 2023-09-21

**Authors:** Zhiwei Jia, Tianlin Wen, Yuning Zhang

**Affiliations:** ^1^Department of Orthopaedics, Dongzhimen Hospital, Beijing University of Chinese Medicine, Beijing, China; ^2^Class 6 of Year 2021 Clinical Medicine, School of Medicine, Shenzhen University, Shenzhen, Guangdong Province, China

**Keywords:** spinal cord injury, axonal regeneration, tanshinone IIA, mechanism, hypothesis

## Abstract

Tanshinone IIA serves as a coenzyme for certain biochemical reactions, exhibiting various pharmacological effects in the treatment of neurological diseases including spinal cord injury (SCI), however, its working mechanism in the treatment of SCI is not clear. Based on previous research, we believe that tanshinone IIA promotes the survival and repair of nerves after spinal cord injury through its pharmacological effects such as anti-inflammatory, antioxidant, and prevention of cellular apoptosis in the spinal cord.

## 1. Introduction

**Figure 1:**
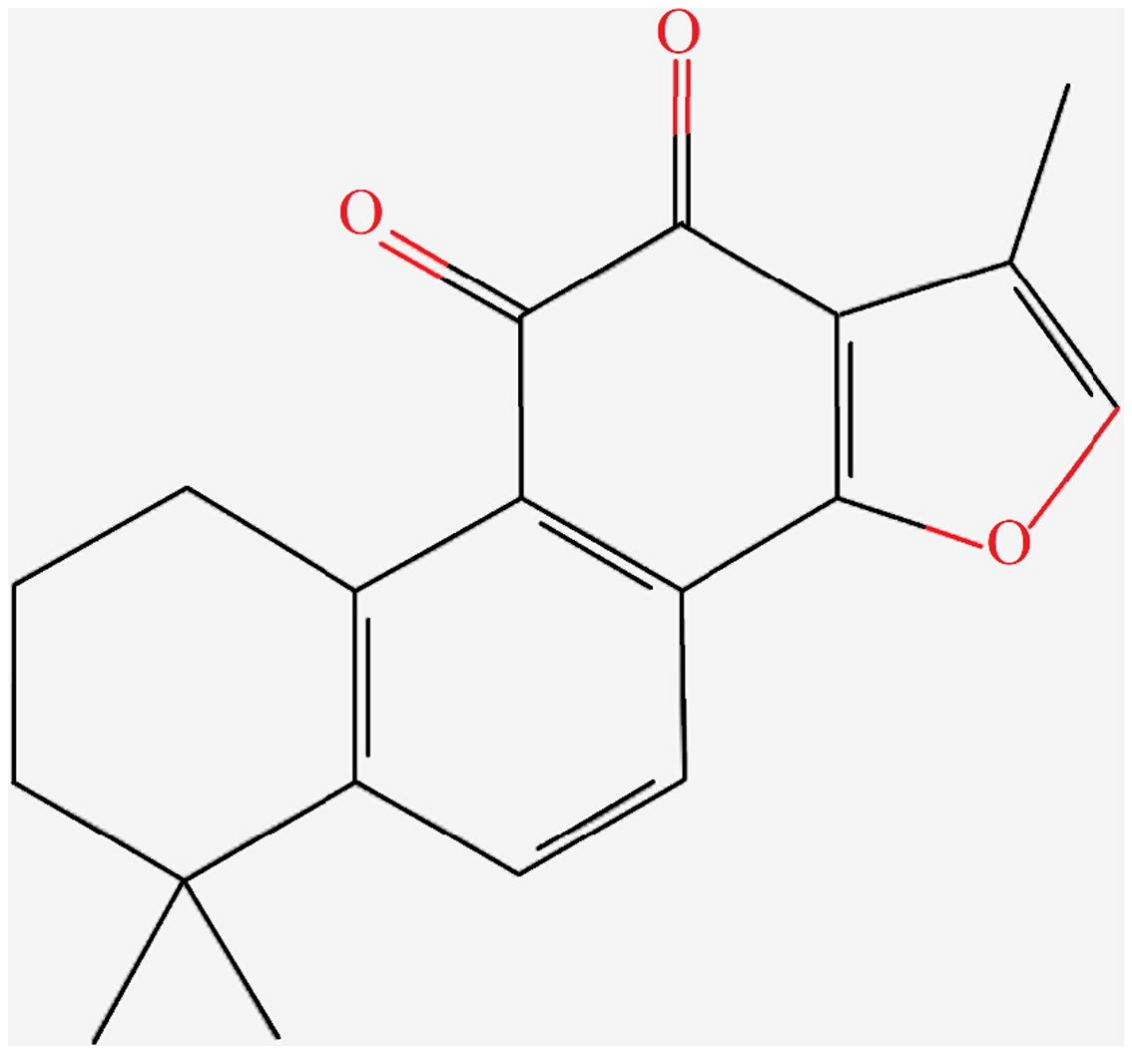
Molecular structure of tanshinone IIA.

Danshen is a perennial herbaceous plant in the family Lamiaceae and the genus Salvia. Tanshinone IIA is the representative lipophilic component of Danshen, which is mainly distributed in the root of the plant. Tanshinone IIA is a cherry red needle-shaped crystal with a molecular weight of 294.34. It is insoluble or slightly soluble in water, and soluble in organic solvents such as dimethyl sulfoxide, ethanol, acetone, ether, and benzene. Supercritical CO_2_ extraction is used to extract tanshinone IIA from Danshen, with the extraction pressure of 25 MPa, extraction temperature of 40°C, extraction time of 2 h, and ethanol flow rate of 1.0 mL/min. The chemical structure of tanshinone IIA was obtained through searching the PubChem Compound database^1^ (Figure 1).

Tanshinone IIA has proven pharmacological effects on cardiovascular diseases, anti-tumor and anti-inflammatory effects, improvement of organ fibrosis as well as neuroprotection. Based on the previous literature research, we hypothesize that, tanshinone IIA promotes the survival and repair of nerves after spinal cord injury through following pharmacological effects:

## 2. Anti-inflammatory effect

Inflammation reaction is the main reason for secondary tissue injury and cellular apoptosis after spinal cord injury. There are studies confirming (Zhang et al., 2010; Yu et al., 2014) that tanshinone IIA has a significant anti-inflammatory effect. It can regulate the production of inflammatory factors IL-6, IL-10, IL-1β, and TNF-α, and weaken the activation of white blood cells. Zhou Yan et al. found that tanshinone IIA can exert anti-inflammatory effects in a mouse model of experimental colitis induced by sodium polysaccharide sulfate by reducing the activity of neutrophils and levels of inflammatory factors (Yan et al., 2017). It can also inhibit the activity of myeloperoxidase and adrenaline in neutrophils, thereby inhibiting joint swelling in mice with gouty arthritis (Xu et al., 2022). Huang et al. (2015) studied the feasibility of tanshinone IIA alleviating cardiac dysfunction, and found that pretreatment with tanshinone IIA significantly attenuates cardiac dysfunction by inhibiting inflammation. Tanshinone IIA was found to alleviate the upregulation of TNF-α and IL-1β induced by LPS, downregulate NADPH oxidase, and reduce phosphorylation levels of extracellular signal-regulated kinase 1/2 (ERK1/2) and mitogen-activated protein kinase (MAPK).

In *in-vitro* studies, tanshinone IIA was reported to inhibit the proliferation of RAW264.7 cells and the production of phospholipase A2, and promote the release of interleukin-10 in RAW264.7 cells, while reducing the production of interleukin-6 (IL-6) and its mRNA expression (Bzour et al., 2011; Fan et al., 2016). In addition, tanshinone IIA can inhibit mRNA expression of TLR4 and TNF-α in EA.hy926 cells stimulated by LPS (Jia et al., 2011), exerting its anti-inflammatory activity. Tang et al. found that tanshinone IIA can inhibit the production of vascular cell adhesion molecule-1 (VCAM-1) and intercellular adhesion molecule-1 (ICAM-1) and dose-dependently inhibit the adhesion of neutrophils to brain microvascular endothelial cells (BMVECs) induced by TNF-α. Tanshinone IIA can reduce the expression of macrophage migration inhibitory factor (MIF) and inflammatory factors (MIF), TNF-α and interleukin (IL)-6 in the brain of rats with cerebral ischemia/reperfusion, thereby alleviating the effects of cerebral ischemia/reperfusion (Chen et al., 2012). Other studies have confirmed that tanshinone IIA can reduce the release of radiation-induced pro-inflammatory cytokines and NF-κBp65 nuclear translocation, and its mechanism of action is to exert its anti-inflammatory properties by inhibiting the transcription of pro-inflammatory cytokine genes associated with the NF-κB signaling pathway, thereby slowing down the progression of Alzheimer’s disease in rats (Jiang et al., 2014). Jiang et al. further found that tanshinone IIA can reduce the production of pro-inflammatory cytokines induced by LPS in RAW264.7 cells and inhibit the phosphorylation of IκB-α in a dose-dependent manner. It can also inhibit the activation of NIK-IKK and MAPKs (p38, ERK1/2, JNK) signaling pathways, thereby inhibiting LPS-induced IκB-α degradation and NF-κB activation to exert anti-inflammatory activity (Jang et al., 2006). Tanshinone IIA was also reported to protect the human blood–brain barrier model from leukocyte-associated hypoxia-reoxygenation injury (Zhang et al., 2010).

Therefore, it is plausible to assume that tanshinone IIA alleviates secondary tissue injury and cellular apoptosis after spinal cord injury by inhibiting inflammatory reaction through TLR4- p38 MAPK and Erk–Jnk–p38-MAPK signaling pathways (Lanna et al., 2017).

## 3. Antioxidation effect

The oxidation stress is also an important cause of further tissue damage after spinal cord injury. It has been confirmed that tanshinone IIA inhibits the generation of DNA adducts and reduces cell toxicity by clearing lipid free radicals and interrupting the chain reaction of lipid peroxidation. It effectively inhibits the interaction between lipid peroxidation products and DNA inside cells, playing a protective role on DNA. Tanshinone IIA can enhance the activity of glutathione peroxidase in brain hypoxic lesions, reduce the content of malondialdehyde, and effectively alleviate oxidative stress (Niu et al., 2000; Tang et al., 2012). Tanshinone IIA was also reported to reduce the concentration of oxidized low-density lipoprotein (OxLDL) in plasma and decrease the generation of superoxide anions and malondialdehyde (MDA) (Tang et al., 2007). In a hypertensive rat model, tanshinone IIA inhibited the activity of NAD(P)H oxidase and the production of reactive oxygen species, exerting antioxidant effects and improving cardiac function (Wang et al., 2011). In both the hydrogen peroxide (H_2_O_2_)-induced J774 macrophage injury model and rat focal cerebral ischemia–reperfusion models, tanshinone IIA significantly increased the activity of glutathione peroxidase (Yu et al., 2008). Research by Wang et al. found that tanshinone IIA can dose-dependently reduce the production of MDA and LDH induced by H_2_O_2_ in human umbilical vein endothelial cells CRL21730, confirming the protective effect of tanshinone IIA from oxidative damage (Weirong and Rong, 2006). Chen et al. confirmed that tanshinone IIA can dose-dependently alleviate apoptosis of human umbilical vein endothelial cells (ECV304) induced by H_2_O_2_, reduce MDA production, increase the activity of superoxide dismutase (SOD), and inhibit the expression of endothelin-1 (ET-1) mRNA. It also increases the expression of peroxisome proliferator-activated receptor gamma (PPAR-γ) mRNA, indicating that tanshinone IIA has a significant protective effect on H_2_O_2_-induced injury in human umbilical vein endothelial cells (Haiming, 2008).

In animal studies, antioxidation effect of tanshinone IIA was used in the treatment of cerebral ischemia–reperfusion injuries 24 h after the onset of the condition. A study by Mingyan et al. (2004) found that tanshinone IIA can reduce the early accumulation of calcium ions ([Ca2+]) after ischemic hypoxic injury. In an experiment conducted by Longshan and Tao (2004), it was discovered that treatment with tanshinone IIA (25 mg·kg-1) significantly reduced the area of cerebral infarction in rats with cerebral ischemia. Tanshinone IIA also counteracted the decrease in superoxide dismutase (SOD) activity and the increase in malondialdehyde (MDA) content induced by cerebral ischemia–reperfusion. In the meanwhile, the molecular pathways for the antioxidation effect of tanshinone IIA are still needed to be further explored.

## 4. Inhibiting cellular apoptosis

Tanshinone IIA can protect neurons from amyloid β protein-induced cytotoxicity by activating cell apoptosis-related pathways (Qian et al., 2012). Tanshinone IIA can enhance the activity of adenosine triphosphatase and protein disulfide isomerase in neurons, improve the balance of energy metabolism, and maintain cellular homeostasis, thus protecting and repairing neurons (Wen et al., 2012). It can also increase the expression of Bcl-2 in ischemic spinal cord, reducing the damage caused by ischemia reperfusion through anti-apoptotic mechanisms (Chen et al., 2012).

In the case of cerebral ischemia, the proliferation and activation of astrocytes lead to the formation of glial scars around necrotic tissue, which can disrupt the normal growth of neuronal axons. Zhou et al. (2015) found that tanshinone IIA can inhibit the activation of astrocytes caused by cerebral ischemia, reduce the formation of glial scars, and thus play a neuroprotective role. Li et al. (2013) found that tanshinone IIA can effectively reduce the expression levels of NF-κB and IκB proteins in the brain tissue of a rat model of cerebral ischemia–reperfusion. Its possible mechanism of action is that tanshinone IIA can inhibit the positive feedback between NF-κB and IκB and reduce the loss of neurons. This is of great significance for further studying the neural protective effect and mechanism of tanshinone IIA administration. Yanhui et al. (2014) used the method of ligation of the abdominal aorta to make a rabbit spinal cord ischemia–reperfusion model. After random grouping, the spinal cord was taken at various time intervals, and venous blood was extracted to detect the glutamate content in the spinal cord and serum. It was found that tanshinone can reduce the local glutamate content in the spinal cord during reperfusion, thereby protecting spinal cord neurons and providing protection against spinal cord ischemia–reperfusion injury in rabbits (Shen et al., 2011).

Based on the above characteristics, tanshinone IIA has been applied to repair neural system injuries. Chen and Shasha (2018) used tanshinone IIA to hippocampal neurons *in vitro* and found that it can protect against radiation damage to the nervous system by reducing cell apoptosis, improving tissue hypoxia conditions, and inducing cell autophagy. In mice with radiation-induced brain damage, tanshinone IIA can inhibit the activation of the ATP-P2X7R axis, suppress the occurrence of inflammatory reactions and accumulation of free radicals, improve spatial learning and memory abilities, and reduce brain edema, thus exerting a neuroprotective effect (Youli et al., 2017). Yin et al. (2012) and Zhong et al. (2021) found that in mice with acute spinal cord injury, tanshinone IIA can decrease the expression of RhoA and ROCKII proteins, inhibit the phosphorylation of myosin light chain, and facilitate the recovery of nerve cells and the regeneration of neuronal axons.

## 5. Conclusion

**Figure 2:**
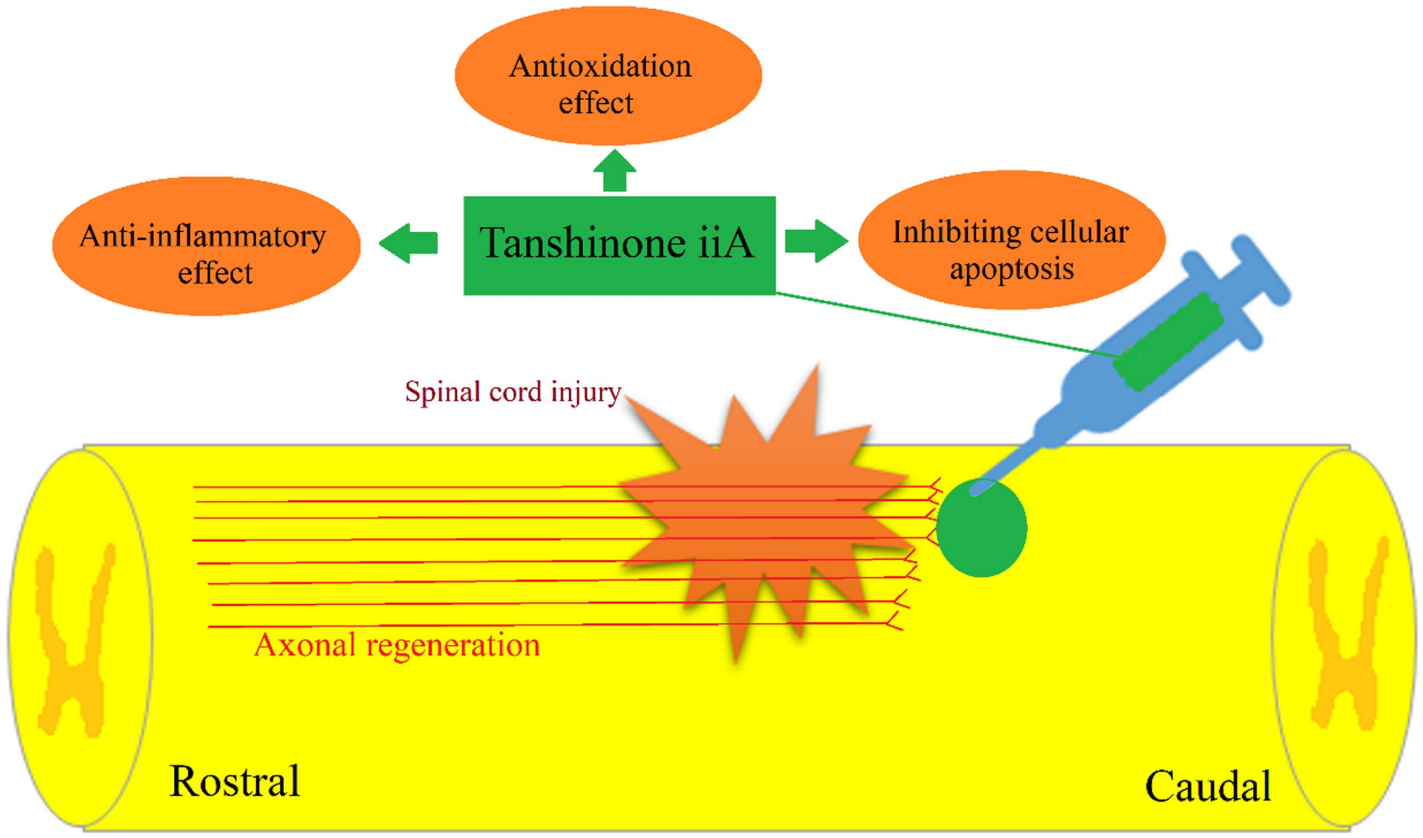
Tanshinone IIA may promote the survival and repair of nerves after spinal cord injury through its anti-inflammatory, antioxidant, anti-apoptosis effects.

Based on the results of its clinical effects, animal and *in vitro* experiments, we believe that tanshinone IIA promotes the survival and repair of nerves after spinal cord injury through its anti-inflammatory, antioxidant, anti-apoptosis effects (Figure 2).

## Data availability statement

The original contributions presented in the study are included in the article/supplementary material, further inquiries can be directed to the corresponding authors.

## Author contributions

ZJ: Conceptualization, Investigation, Writing – original draft. TW: Funding acquisition, Supervision, Validation, Writing – review & editing. YZ: Investigation, Writing – original draft.

## Funding

The author(s) declare financial support was received for the research, authorship, and/or publication of this article. The current research is supported by the National Natural Scientific Foundation of China (nos: 82074452, 82260252, and 81860235).

## Conflict of interest

The authors declare that the research was conducted in the absence of any commercial or financial relationships that could be construed as a potential conflict of interest.

The handling editor AM declared a past co-authorship with the author TW.

## Publisher’s note

All claims expressed in this article are solely those of the authors and do not necessarily represent those of their affiliated organizations, or those of the publisher, the editors and the reviewers. Any product that may be evaluated in this article, or claim that may be made by its manufacturer, is not guaranteed or endorsed by the publisher.

## Footnotes

^1^ http://www.ncbi.nlm.nih.gov/pccompound
